# Additive Manufacturing Processes: Selective Laser Melting, Electron Beam Melting and Binder Jetting—Selection Guidelines

**DOI:** 10.3390/ma10060672

**Published:** 2017-06-19

**Authors:** Prashanth Konda Gokuldoss, Sri Kolla, Jürgen Eckert

**Affiliations:** 1 Department of Manufacturing and Civil Engineering, Norwegian University of Science and Technology, Teknologivegen 22, 2815 Gjøvik, Norway; sri.kolla@ntnu.no; 2 Erich Schmid Institute of Materials Science, Austrian Academy of Sciences, Jahnstraße 12, 8700 Leoben, Austria; juergen.eckert@oeaw.ac.at or juergen.eckert@unileoben.ac.at; 3 Department of Materials Physics, Montanuniversität Leoben, Jahnstraße 12, A-8700 Leoben, Austria

**Keywords:** additive manufacturing, selective laser melting, electron beam melting

## Abstract

Additive manufacturing (AM), also known as 3D printing or rapid prototyping, is gaining increasing attention due to its ability to produce parts with added functionality and increased complexities in geometrical design, on top of the fact that it is theoretically possible to produce any shape without limitations. However, most of the research on additive manufacturing techniques are focused on the development of materials/process parameters/products design with different additive manufacturing processes such as selective laser melting, electron beam melting, or binder jetting. However, we do not have any guidelines that discuss the selection of the most suitable additive manufacturing process, depending on the material to be processed, the complexity of the parts to be produced, or the design considerations. Considering the very fact that no reports deal with this process selection, the present manuscript aims to discuss the different selection criteria that are to be considered, in order to select the best AM process (binder jetting/selective laser melting/electron beam melting) for fabricating a specific component with a defined set of material properties.

## 1. Introduction

Additive manufacturing (AM), also known as 3D printing or rapid prototyping, is gaining increased attention and interest due to the substantial increase in the demand for high performance materials with added functionalities (such as internal cooling channels or internal lattice structures, which are difficult to fabricate with conventional manufacturing processes) and increased complexities in geometrical design [1,2,3]. AM processes possess the capability of producing 3D parts with near-net-shaped dimensions layer-by-layer directly from 3D computer-aided design data [4,5]. Some of the AM processes require no post-processing or minimal post-processing, and the produced parts can be directly used in real-time applications [6,7,8]. With such advantages, AM processes have found application in almost all fields ranging from aerospace, automotive, medical, machinery, marine/oil and gas, and electronics industries, to consumer applications (jewelry/fashion apparel/phone accessories), building construction, and the food industry, etc. [9,10,11].

There are several AM processes available for the fabrication of metals, such as binder jetting (BJG), the powder bed fusion process (selective laser melting (SLM) and electron beam melting (EBM) processes), metal extrusion, sheet lamination, direct energy deposition etc. Of these, binder jetting and the powder bed fusion processes use metal powders as raw materials [12,13,14,15,16]. The other processes, such as metal extrusion, use wires/rods [17,18]; sheet lamination use sheets; and the direct energy deposition process usually involves wires as the material source. However, some of these processes may also involve powder as a raw material source [19,20,21,22]. Each of these AM processes has their own pros and cons. For instance, powder bed fusion processes like SLM has high cooling rates, varying between ~10^4^ and 10^6^ Ks^−1^ [1,23,24,25]. In addition, the parts fabricated by SLM tend to show improved mechanical, tribological, and corrosion properties compared to their cast counterparts [26,27,28,29,30].

On the other hand, processes like EBM use a hot bed (>870 K) and hence do not produce a fine microstructure like the SLM process [1,31,32]. Powder bed fusion processes like SLM and EBM have similar working principles, where both employ layer-by-layer technology with the fusion of powder particles through a beam-electron/laser. Yet, there exist some differences between the two processes, as SLM operates under an inert atmosphere with a cold powder bed while EBM operates under vacuum and generally with a hot powder bed, which hence affects both the quality and properties of the fabricated parts. BJG contains several processing steps (including the actual deposition, de-binding, curing, sintering, HIPing etc.), unlike the EBM and SLM processes. The working principle of the BJG process is completely different from that of the powder bed fusion processes (EBM and SLM), as no melting of powders take place. Fusion between the adjacent powder particles is due to the applied thermal energy, which follows the conventional sintering mechanisms [33,34,35,36,37]. The properties of the parts produced by BJG will be different from those of SLM and EBM, because BJG works with a conventional sintering mechanism. On the other hand, SLM and EBM achieve a significantly finer microstructure due to faster cooling rates than the BJG process. Most importantly, the types of materials/alloy systems used by BJG, SLM, and EBM differ, due to the differences in their working principles. Hence, it is important to select the right AM process for the manufacturing of specific materials. However, there are no reports available that give guidelines on selecting the right AM process depending on the material to be processed, properties required for the parts, etc. Therefore, the present manuscript aims to provide some guidelines (based on the material to be processed, availability of technology, properties and service requirement, post-processing requirements, surface quality of the parts, and the accuracy requirements of the manufactured parts) that may be helpful in selecting the best AM process among BJG, SLM, and EBM.

## 2. Additive Manufacturing Processes

### 2.1. Binder Jetting

BJG is one of the multistep AM processes originally developed at Massachusetts Institute of Technology (MIT) in the early 1990s [34,38]. Even though it was developed in the 90s, it was a considerable time until its commercialization in 2010 [38]. This technology has the capability of handling metals/alloys (including Al-based, Cu-based, Fe-based, Ni-based, and Co-based alloys) and ceramics (including glass, sand, graphite, etc.). However, it is said to work with any material that is available in the form of powder and allows color printing. The BJG process normally uses two materials, namely the metal/ceramic-based material of which the part is to be made and a binder material, which glues the metal/ceramic powder material between and within the layers. The binder is usually a liquid and the metal/ceramic is in the form of a solid powder. The printing process is similar to any other printing process that takes places in the manufacturing of an AM part. The metal/ceramic is spread and a layer of binder is deposited over the powder metal/ceramic layer, where required, which is dictated by the Computer Aided Design (CAD) model. This process is iterated for building the entire part. However, the BJG process involves several post-processes that follow the printing of the parts such as curing, de-powdering, sintering, infiltration, annealing, and finishing [39,40]. These post-processes sometimes take longer time than the actual printing (especially the sintering of the parts) and may incur significant costs. One of the significant advantages of BJG is that the parts can be produced without support structures. The build parts lie on the loose powder bed, which is not bonded together. Hence, the entire build volume can be stacked with several parts with a gap of just few layers of distance between them [41].

Since BJG use binders as adhesive, the material characteristics are not always suitable for structural applications for aerospace and automobile parts, since they may lead to porosity as in the conventional sintering process. The printing process itself is faster than SLM/EBM processes and can be accelerated by increasing the number of print head holes that deposit the material and the binder. It also allows a two-material approach, where different powder-binder combinations can lead to different mechanical properties, simply by changing the powder-binder ratio. Coarse powders can also be used in this process, which significantly cuts the cost of manufacturing very fine powders. As these methods do not involve heating during the building process, there are no residual stresses created in the parts, unlike in the SLM process, and BJG is regarded as one of the most cost-effective AM processes to build three-dimensional parts with added functionalities [42,43,44]. Since there is no melting involved in the BJG process and the consolidation takes place predominantly by sintering, there is always a possibility for the presence of porosities, and the volume, size, and shape of the pores may differ within different parts produced in the same batch [44]. Moreover, the parts are expected to have a coarse microstructure, since the parts have to undergo thermal treatments such as curing, sintering, and annealing once they are printed with the binder. Hence, the mechanical properties of BJG parts are not as strong as the parts produced by SLM/EBM.

### 2.2. Selective Laser Melting

SLM is one of the powder bed fusion processes, which are the most widely used in the AM industry [1]. As the name suggests, SLM uses a laser beam that melts and fuses the metal powders together. Similar to the BJG process, a thin layer of powder is deposited over a substrate plate or on the previously deposited layer and the laser beam melts and fuses the powder particles selectively, as dictated by the CAD data [45,46,47]. Several process parameters have to be tuned carefully in order to fabricate a defect-free part [48,49,50]. Some of the important process parameters are laser power, laser scan speed, hatch distance, hatch overlaps, hatch style, etc., which also have a significant effect on the mechanical properties of the parts [23,27]. The entire process takes places inside a closed chamber, usually filled with an inert gas like N2 or Ar, depending on the reactivity of the metal powder to be used. In addition, the build chamber is subjected to over pressure conditions. The presence of an inert gas and over pressure conditions in the chamber minimizes the oxygen contamination during the process. There is always a possibility to use a substrate plate heating (200–500 °C) in order to minimize the cooling rate, if desired [13]. Substrate plate heating is generally employed during the processing of brittle and high temperature materials to reduce the cooling rate, in order to prevent possible cracking during solidification [13].

SLM is regarded as the most versatile AM process, because it can process a wide spectrum of materials including Al-based alloys [51,52,53], Ti-based alloys [54,55,56], Fe-based alloys [57,58,59], Ni-based alloys [60,61,62], Co-based alloys [63,64,65], Cu-based alloys [6], and their composites [66,67,68,69]. Moreover, reports also show that SLM is capable of producing amorphous materials [24,70], because of the high cooling rates observed during the process [23,24,25]. Recent reports show that the mechanical properties of the alloy can also be tuned depending on the requirement, by varying the process parameters during the process (such as hatch style variations, contour variation, base plate heating, internal heat treatment, etc.), which in turn has an influence on the final microstructure of the parts [27]. The process is relatively slow, compared to the BJG process; however, multiple laser sources can be introduced to improve the building rate of the SLM process. Studies have shown that the powders can theoretically be reused repeatedly [71]. This reduces the wastage of raw materials and hence leads to a greener environment.

Some of the biggest advantages of using SLM as the AM process are: the use of a large range of materials, the ability to tune properties during the processing of the parts, increased functionality, relatively low cost, and the production of near-net-shaped components ready to use (if the surface roughness levels are acceptable). On the other hand, SLM may have the following draw backs: it has a relatively slow process (because of the process speed limitations), acute size restrictions, high power usage, high initial costs, the optimization of the process parameters is time consuming, the powder handling can be tricky, and the produced parts may have rough surfaces (depending on the powder size and the process parameters). In addition, brittle materials and high temperature materials that cannot accommodate high internal stress during the fabrication process will lead to cracking of the parts, which to a certain extent can be overcome by reducing the cooling rate (by employing substrate plate heating). At the same time, it may also lead to anisotropic microstructure in the material along the building direction [27].

### 2.3. Electron Beam Melting

EBM is very similar to the SLM process, which works on layer-by-layer technology. However, the EBM process has some differences when compared to the SLM process. An electron beam is used for the melting and fusion of the powder particles instead of a laser beam. The powder bed is kept at high temperatures (>870 K) and overnight cooling times are required to cool the powder bed after the completion of the build job. The EBM process involves more process parameters, including: beam power, beam scanning velocity, beam focus, beam diameter, beam line spacing, plate temperature, pre-heat temperature (including the repetitions, speed, and power of the beam), contour strategies, and scan strategy. The optimization of the process parameters is even more difficult than the SLM process and hence only limited materials are employed in EBM (Ti grade 2, Ti6Al4V, Inconel 718, CoCrMo) [72]. The process is rather slow and it makes the parts very expensive. Additionally, restrictions exist in terms of both the size of the parts and the minimum size of a cell in a lattice structure/honeycomb. Nevertheless, parts with sizes bigger than the substrate plate can be built. However, the size of a part’s initial layers should be less than the size of the substrate plate. The EBM process takes place under a vacuum atmosphere, unlike the inert atmosphere during the SLM process. Hence, oxidation of the parts is generally averted. In addition, any adsorbed gases along the surface of the powder particles will not lead to the formation of porosity in the EBM process. However, it is not advisable to process alloys that have volatile constituents such as Zn, Mg, Pb, Bi, etc.

EBM has the capability of processing brittle materials that generally cannot be processed by SLM. Brittle materials like the intermetallics are generally expected to have poor thermal expansion and contraction behaviors. When these materials are cooled at a very fast rate from their melting points/solidifying ranges, they solidify quickly yet at the same time they cannot accommodate the internal stresses as a result of solidification process, which hence leads to the formation of cracks, also known as solidification cracks. Since the SLM process generally employs high cooling rates, brittle materials exhibit the formation of solidification cracks. On the other hand, in case of the EBM process, the cooling rate of the process can be reduced drastically by increasing the temperature of the powder bed. Generally, the hot bed temperature is around 870 K during the EBM process. Under such conditions, a very slow cooling of the melt takes place and solidification cracking in brittle materials can be avoided. Hence, brittle materials like the intermetallics (TiAl) and high entropy alloys can be processed by the EBM process without the formation of solidification cracks, by carefully choosing the temperature of the powder bed. The electron beam may be used multiple times to heat the powder bed and then to melt the parts selectively. Since the electron beam is used multiple times in each layer, the time taken to process each layer is much higher than the time needed in the SLM process. In addition, the entire chamber becomes so hot after the building process that it may require considerable cooling time before the parts can be removed from the substrate plate. In general, overnight cooling is necessary before the powder bed reaches room temperature and the parts can be removed from the chamber and the substrate plate.

## 3. Process Selection Considerations

**Figure 1:**
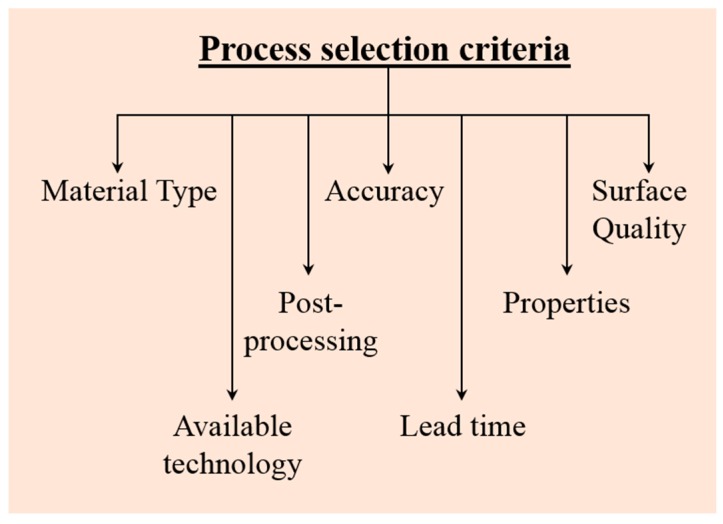
Schematic illustration showing the different factors that should be considered in selecting the right additive manufacturing process (binder jetting, selective laser melting, or electron beam melting).

First step in our selection process is to check whether there is any advantage of using AM for particular parts. There are some guidelines that have to be followed to verify if it is beneficial to fabricate a part using one of the AM processes. We are not discussing the criteria here, since that is out of the scope of the present manuscript. However, once it is determined that AM is required to manufacture a particular part, several factors are to be taken into account in order select the right AM process (BJG/SLM/EBM) for fabrication (Figure 1).

### 3.1. Type of Material to be Processed and Their Properties

The type of material to be fabricated plays an influential role in deciding the type of AM process to be used. For instance, for the processing of ceramic materials, the best-suited process among the three is the BJG process, which will be a straightforward process selection. On the other hand, when it comes to metals/composites, all the three processes of BJG, SLM and EBM compete with each other. However, the selection has to be made based on the properties of the material along with the other selection criteria. For instance, consider the fabrication of Mg/Zn-based materials. EBM may not be the most suitable process, since the powder bed is held at high temperatures (more than 870 K), where some of the materials melt. Moreover, very intense energy will be supplied to the powder bed by the electron beam, which has the capability of vaporizing Mg/Zn, since they have very low boiling points. Not only is EBM is a bad process choice for Mg/Zn-based materials, but it also would result in the contamination of the chamber due to the deposition of these materials inside the build chamber (once they vaporize from the powder bed). Hence, EBM is not suitable for the fabrication of Al/Mg/Zn-based materials. Both BJG and SLM stake their claims for the processing of Mg/Zn-based alloys.

Consider the fabrication of brittle materials such as TiAl or hard intermetallics, where all three AM methods, BJG, SLM and EBM, can theoretically process these materials. However, the extremely high cooling rates observed during the SLM process (between ~10^4^ and 10^6^ Ks^−1^ [23,24,25]) may lead to high internal stresses in the hard intermetallics [13,73]. These intermetallics cannot accommodate such high internal stress, which will lead to the formation of cracks perpendicular to the scanning direction [73]. Such cracks can be eliminated/minimized by reducing the cooling rate of the solidification process. This can be achieved by employing substrate plate heating. The substrate plate heating temperature depends on the material to be fabricated. For instance, for Al-based alloys, temperatures between 473 and 673 K should be sufficient to eliminate these cracks [13]. However, for materials like TiAl, higher substrate plate temperatures are required (in the range of 773–973 K), which makes the fabrication of materials like TiAl difficult using the SLM process. Under such circumstances, BJG or EBM may offer alternative processing routes. High entropy alloys (HEA) are similar to TiAl; they are generally considered to be brittle. In addition, HEAs contain more than three or four elements in equi-atomic configuration with a range of melting points, which make them difficult to process by any fusion method. There are some reports that deal with the processing of HEAs by SLM and EBM, however, they are not widely used due to the difficulties involved. BJG is the obvious choice, since it minimizes the process complications (related to alloying elements with a wide melting range, differences in thermal conductivities between the alloying elements, difference in the coefficient of thermal expansion between the alloying elements, etc.) observed in the SLM/EBM processes.

Super high strength materials like diamond, which also has a high melting point, cannot be effectively processed by EBM or SLM; hence, BJG is the only alternative. In addition, the processing of diamond using SLM/EBM may initiate a phase transformation in diamond that is not desired. Consider the processing of glassy/amorphous materials, where EBM cannot be chosen because of the high temperature of the powder bed, which will crystallize the amorphous parts. Similarly, BJG cannot be used to process amorphous alloys, because the de-binding and sintering process involves thermal treatments for longer periods that are sufficient to crystallize the amorphous precursors. Hence, SLM can be the only option with sufficient control of the cooling rates to process such amorphous precursors.

### 3.2. Technological Limitations

Consider the fabrication of 316L (Stainless steel) or 1.2709 (maraging steel). Theoretically, all three processing methods, BJG, SLM and EBM, should work. However, for the processing of 316L and 1.2709 steels with EBM, there are no defined industrial process parameters available to produce a defect-free component. Hence, such limitations in the available technology restricts the fabrication of 316L/1.2709 by the EBM process. However, extensive research may be carried out to optimize the processing conditions and parameters for such materials, which involve extensive costs and resources. Similarly, consider the fabrication of Ti-based alloys like the commercially pure Ti or Ti-6Al-4V, or the fabrication of Inconel 625/718 or CoCrMo; all the three processes can fabricate these types of materials, thus other criteria should be considered for the selection of the right manufacturing process.

### 3.3. Materials Properties—Service Requirements

The properties required for specific service conditions also play a role in choosing the fabrication process. For instance, a recent report showed that the tensile properties of Al-12Si samples produced by SLM can be tuned, where the yield strength can be varied between 235 and 290 MPa, the ultimate strength can be varied between 220 and 460 MPa, and the ductility can be varied between 2.8% and 9.5%, respectively, in tension [27]. These property variations are imparted in the material by varying the microstructure directly during the fabrication process; by changing the process parameters and the processing strategy [27]. However, neither the EBM nor the BJG processes offer such variation in mechanical properties. Similarly, Ti6Al4V alloys produced by SLM or EBM show different mechanical behaviors. For instance, vertically built Ti6Al4V samples show a yield strength of ~870 MPa, ultimate strength of ~928 MPa, and ductility of ~10% in tension, when fabricated by EBM. However, the same material processed by SLM show a yield strength of ~1140 MPa, ultimate strength of ~1220 MPa (which is 31% higher than the sample built with the EBM process), and ductility of ~5% (50% less ductility than the material built with the EBM process) [74]. This is due to the fact that the SLM process has high cooling rates and ends up with a martensitic structure, which improves the strength of the material at the expense of ductility. On the other hand, the hot powder bed in the EBM process avoids the formation of brittle martensite in Ti6Al4V samples, and hence they have better ductility with slightly lower strength levels. Similarly, the samples produced by the BJG process will have inferior strength levels compared to the samples prepared by either SLM or EBM, because of prolonged thermal treatments (curing, sintering, annealing, etc.). Hence, the material properties required for particular service conditions influence the selection of the fabrication process (among BJG/SLM/EBM). Consider the fabrication of 316L stainless steel by both BJG and SLM processes. As expected, the SLM 316L will exhibit better mechanical properties than the BJG 316L. Also, the BJG 316L may have significant porosities in it, compared to the SLM sample, depending on the processing conditions [75,76]. Hence, when the material has to be used for structural applications, the SLM process is preferred over the BJG process, because of the strength requirements and defect levels (porosity distribution in the sample).

### 3.4. Post-Processing

Similarly, if the same 316L is used for functional, electrochemical, or biomedical applications, where strength is not an issue and the presence of internal porosity might be beneficial during its service, then the obvious choice for fabrication is the BJG process, since it is cheap and satisfies all of part’s the requirements. SLM/EBM are related as technologies that can produce near-net-shaped components [4,5]. Consider, if a part that is fabricated using SLM/EBM has to undergo significant post-processing such as electro-polishing, external thermal treatment, or the application of surface coatings, then BJG also comes as a strong competitor, since one of the major drawbacks of BJG is considered to be the series of post-processing operations necessary after the actual printing of the parts. In addition, if the parts are considerably big and cannot fit in the chamber of SLM/EBM, then the only option to choose is BJG. Hence, both size and shape restrictions along with the post-processing requirements play a significant role in choosing the best fabrication process for a given component.

### 3.5. Surface Quality and Tolerance Levels

Surface quality is another concern with AM parts. The parts produced by SLM are rough, if large sized (30–120 µm) powder particles are used for the fabrication process. However, theoretically it will be better to use particles with a size of 20 µm or less in order to have a smooth surface finish on the manufactured parts. Yet, the powder with smaller particles size (20 µm or less) will severely hamper the flowability of the powder and hence is not desired, because it fails to spread as a uniform layer on the substrate [77]. In addition, the production of powder with small particles size will increase the production costs of the powder. Similarly, the surface quality of the parts is also a concern in the EBM process. On the other hand, the BJG process can use powder particles of any size (theoretically including 20 µm or less) and hence may lead to better surface quality of the parts. This shows that the surface quality requirements also play an important role in deciding the right fabrication process.

The parts produced by BJG need significant tolerance levels. This is primarily because of the extensive thermal treatments involved in the BJG fabrication process. For instance, the prolonged sintering process may lead to distortion or significant dimensional changes [78,79,80]. Hence, in order to overcome the distortion or dimensional changes during the thermal treatments, additional material allowances should be given to the parts during the production stage, which can later be machined off to exact dimensions. Such thermal treatments involve additional post-processing and materials usage in order to have parts with exact dimensions. On the other hand, the SLM/EBM processes produce parts with accuracy (theoretically), or only minimum tolerances are required. When there are strict requirements for the dimensions of the parts, the SLM/EBM processes are preferred over the BJG process. Lead-time is another criterion, which has to be considered because processes like EBM take considerable time for the chamber to cool down after the fabrication process, and with BJG the process chain itself is too time consuming, unlike the SLM process. Since the different AM processes have different process chains and times required for fabrication, the lead-time becomes another very important factor to be considered. There are also other factors that may play a role in the selection process, such as (1) the availability of powder according to size requirements; (2) the number of parts to be produced and the available parameter sets; (3) the design complexities; and (4) the resources available. However, these criteria are not as important as the above-discussed criteria including the type of material to be processed, the available technology, and the property requirements of the part in service.

## 4. Summary

According to the focus of the manuscript (AM process selection among BJG, SLM, and EBM), we have devised some selection criteria that are to be considered in order to select the best AM manufacturing process (BJG/SLM/EBM) for fabricating a particular component with a defined set of material properties. They are: (1) the type of material to be processed; (2) the technology availability; (3) the properties and service requirement of the parts; (4) the application of the parts; (5) the post-processing requirements; (6) the surface quality of the parts; and (7) the accuracy of the parts. Several other parameters are involved that can also be considered for the selection of a suitable AM process. However, these seven criteria we considered to be the most important, hence, they are discussed in detail with examples.
